# Supplementary figures and images for: Metformin regulates myoblast differentiation through an AMPK-dependent mechanism
Source: PLoS One. 2023 Feb 10;18(2):e0281718. doi: 10.1371/journal.pone.0281718 (PMC9916624; doi:10.1371/journal.pone.0281718)

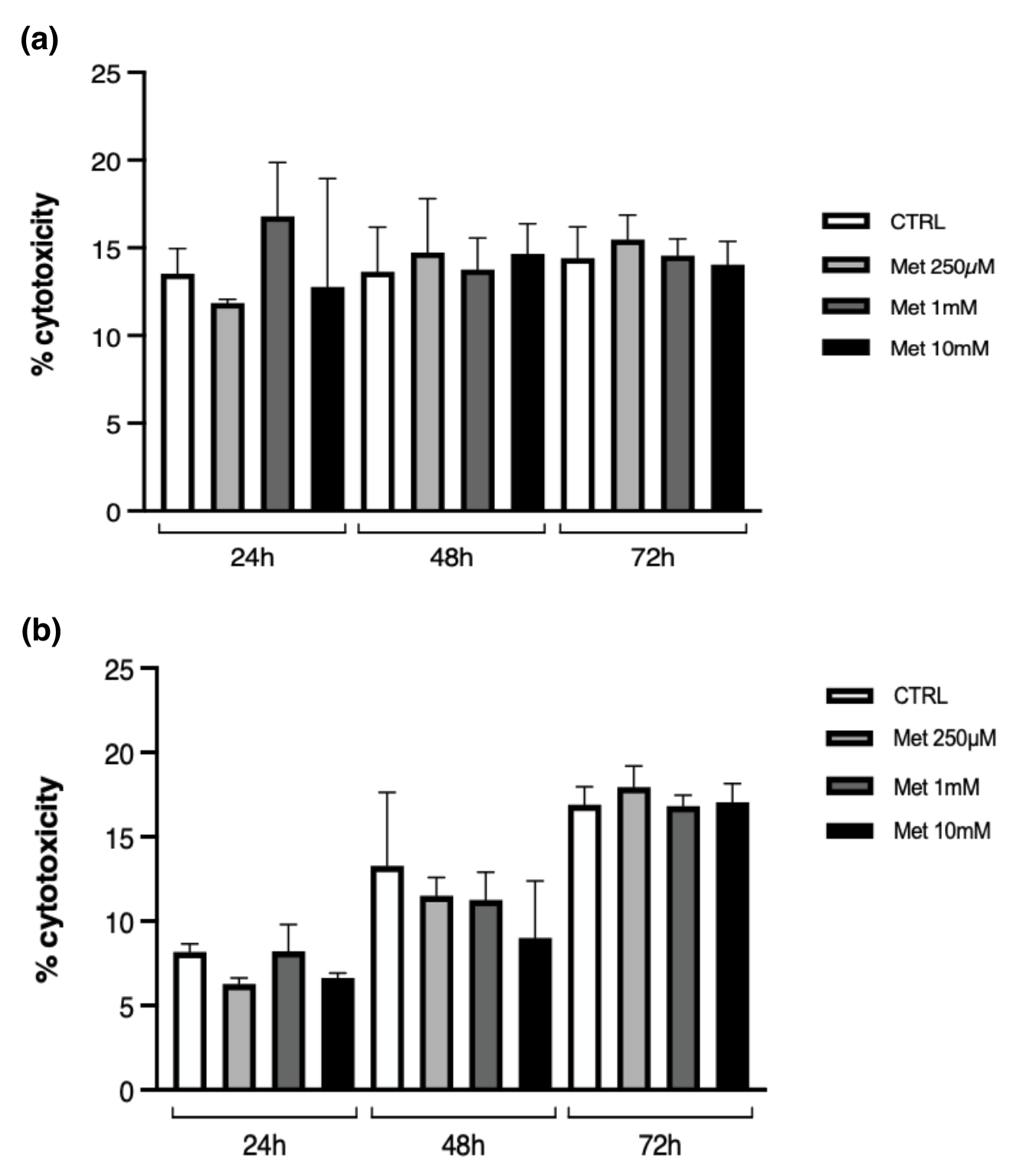

Supplement: S1 Fig — TB exclusion test (a) and LDH release assessment (b) were performed. Statistical analysis of the differences between control and treated cells was conducted for each time point using one-way ANOVA. No differences were observed (p>0.05). (TIF) [file pone.0281718.s001.tif]

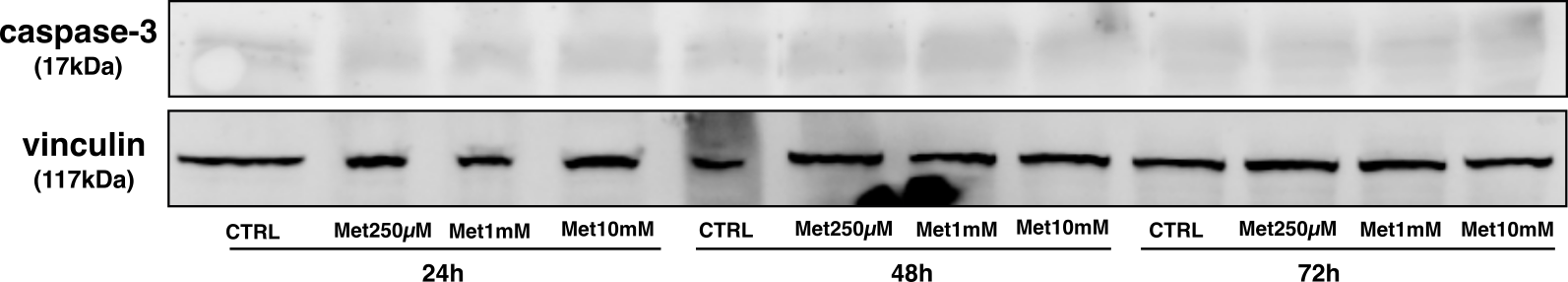

Supplement: S2 Fig — WB analysis revealed no differences in caspase-3 expression in Met treated myoblast at any time point and concentration. (TIF) [file pone.0281718.s002.tif]
