# Supplementary material for: Metformin regulates myoblast differentiation through an AMPK-dependent mechanism
Source: PLoS One. 2023 Feb 10;18(2):e0281718. doi: 10.1371/journal.pone.0281718 (PMC9916624; doi:10.1371/journal.pone.0281718)
Supplement: S1 Raw images — (PDF) [file pone.0281718.s003.pdf]

Identity of experimental samples: C2C12 total lysates.
Image captured by using ChemiDoc™ Touch Image System (Bio-Rad).
Panel generated from this original image: AMPK of Fig 2.

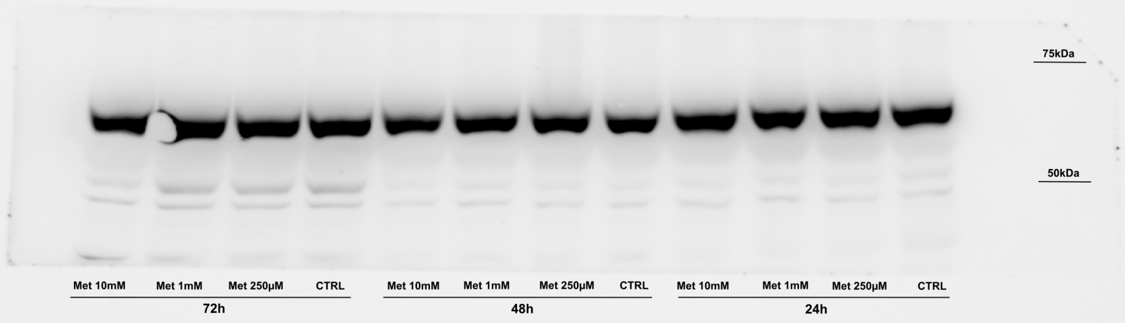

Identity of experimental samples: C2C12 total lysates.
Image captured by using ChemiDoc™ Touch Image System (Bio-Rad).
Panel generated from this original image: p-AMPK of Fig 2.

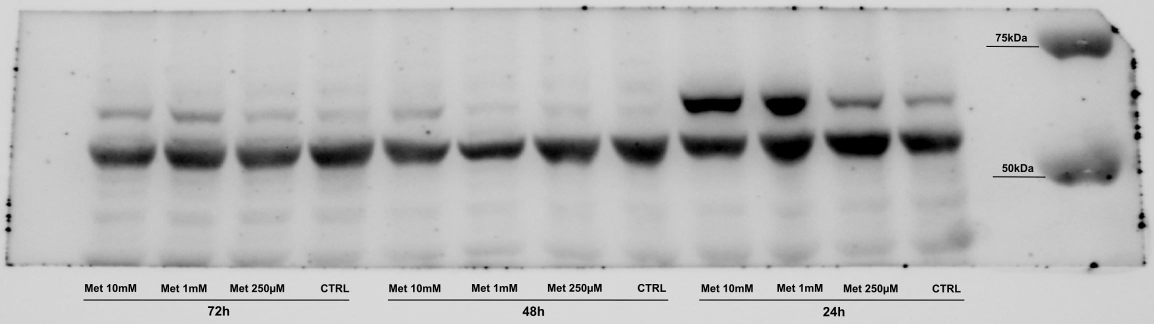

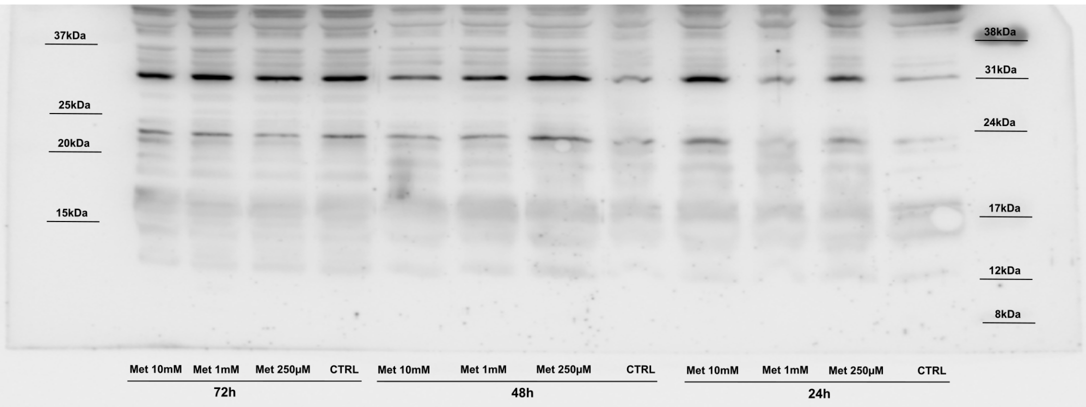

Identity of experimental samples: C2C12 total lysates.
Image captured by using ChemiDoc™ Touch Image System (Bio-Rad).
Panel generated from this original image: caspase-3 of Fig S2.

Identity of experimental samples: C2C12 total lysates.
Image captured by using ChemiDoc™ Touch Image System (Bio-Rad).
Panel generated from this original image: vinculin of Fig S2.

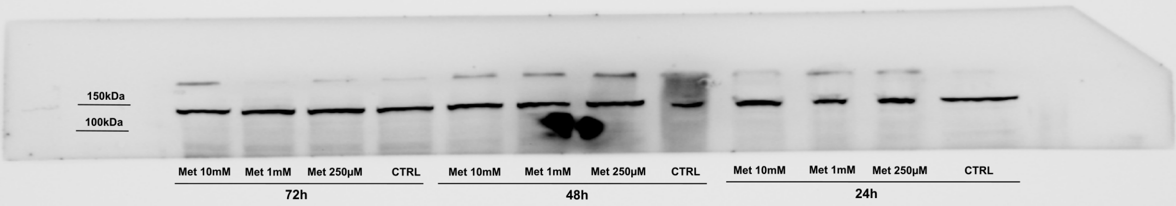

Identity of experimental samples: C2C12 total lysates.
Image captured by using ChemiDoc™ Touch Image System (Bio-Rad).
Panel generated from this original image: MYH1/2 of Fig 4a.

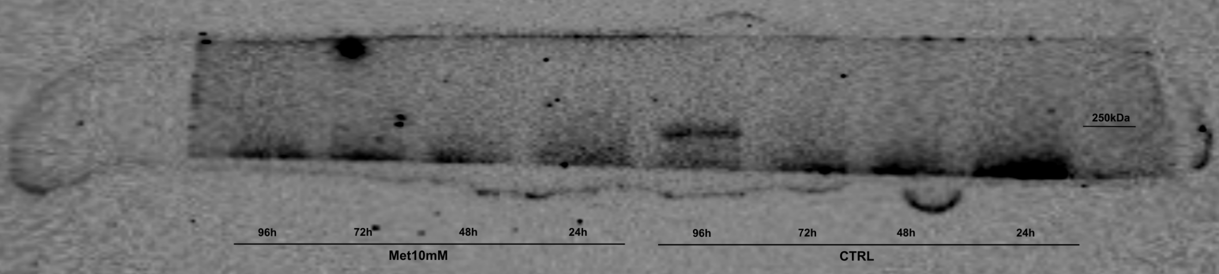

Identity of experimental samples: C2C12 total lysates.
Image captured by using ChemiDoc™ Touch Image System (Bio-Rad).
Panel generated from this original image: GAPDH of Fig 4a.

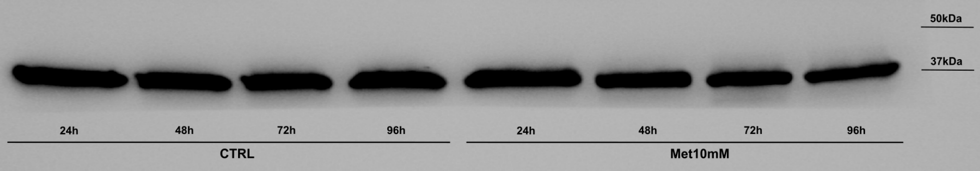

Identity of experimental samples: C2C12 total lysates.
Image captured by using ChemiDoc™ Touch Image System (Bio-Rad).
Panel generated from this original image: PAX7 of Fig 4a.

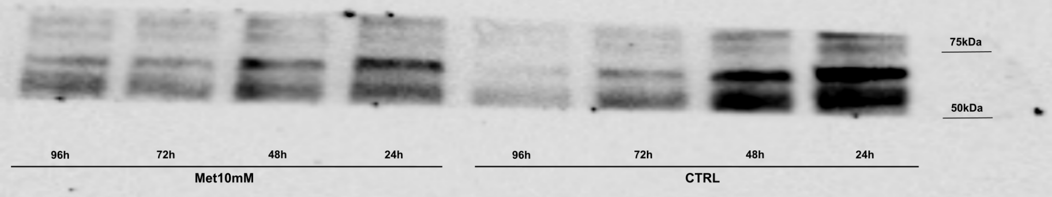

Identity of experimental samples: C2C12 total lysates.
Image captured by using ChemiDoc™ Touch Image System (Bio-Rad).
Panel generated from this original image: Myf5 of Fig 4a.

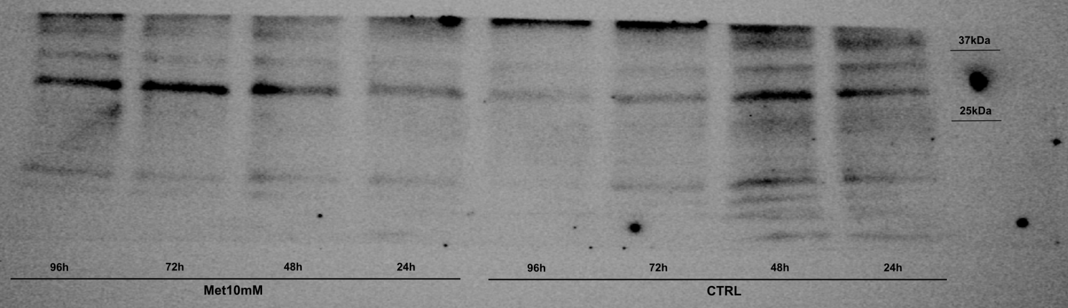

Identity of experimental samples: C2C12 total lysates.
Image captured by using ChemiDoc™ Touch Image System (Bio-Rad).
Panel generated from this original image: p21 of Fig 4a.

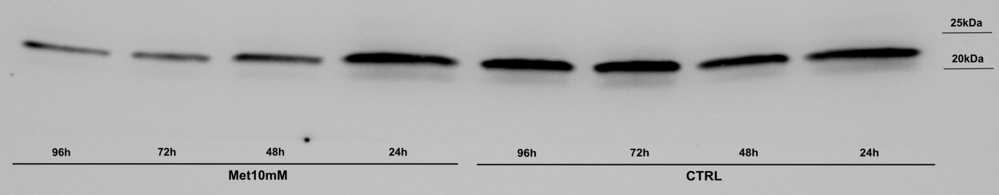

Identity of experimental samples: C2C12 total lysates.
Image captured by using ChemiDoc™ Touch Image System (Bio-Rad).
Panel generated from this original image: vinculin of Fig 4a.

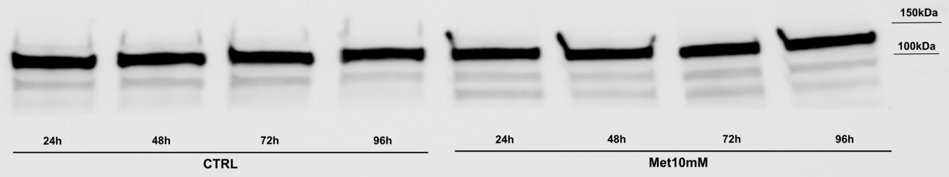

Identity of experimental samples: C2C12 total lysates.
Image captured by using ChemiDoc™ Touch Image System (Bio-Rad).
Panel generated from this original image: MyoD of Fig 4a.

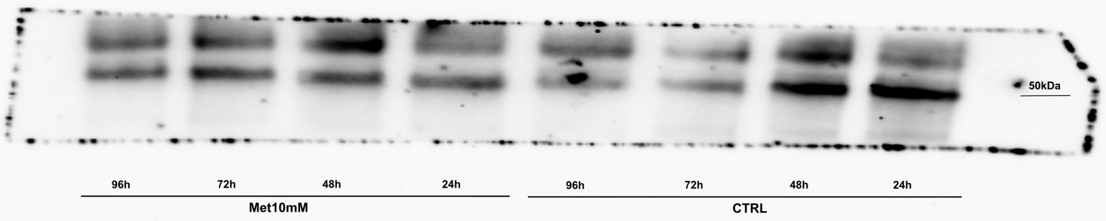

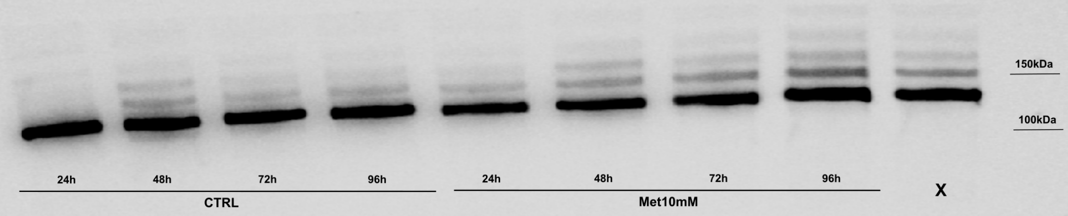

Identity of experimental samples: C2C12 total lysates.
Image captured by using ChemiDoc™ Touch Image System (Bio-Rad).
Panel generated from this original image: vinculin of Fig 4a.

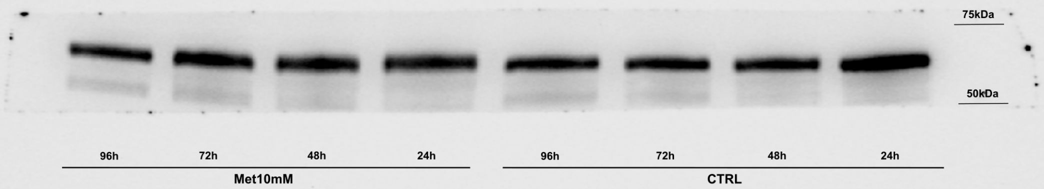

Identity of experimental samples: C2C12 total lysates.
Image captured by using ChemiDoc™ Touch Image System (Bio-Rad).
Panel generated from this original image: AMPK of Fig 5a.

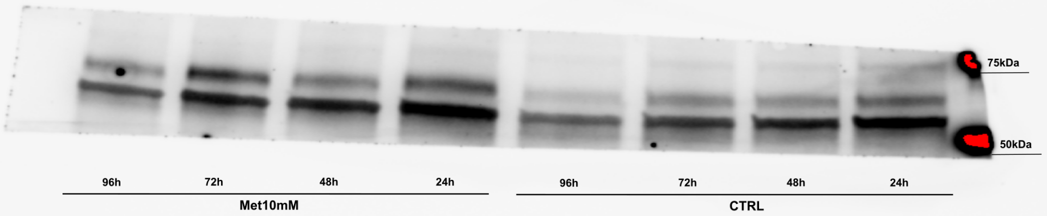

Identity of experimental samples: C2C12 total lysates.
Image captured by using ChemiDoc™ Touch Image System (Bio-Rad).
Panel generated from this original image: p-AMPK of Fig 5a.

Identity of experimental samples: C2C12 total lysates.
Image captured by using ChemiDoc™ Touch Image System (Bio-Rad).
Panel generated from this original image: PGC1α of Fig 5b.

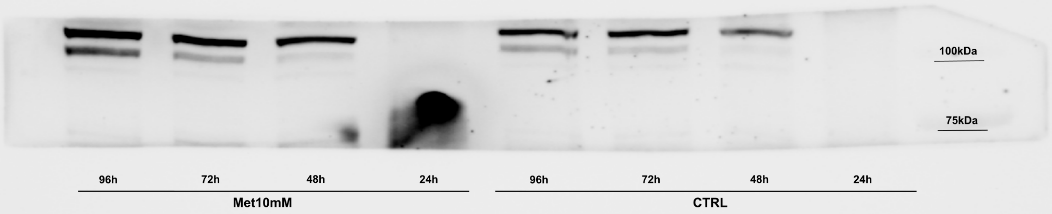

Identity of experimental samples: C2C12 total lysates.
Image captured by using ChemiDoc™ Touch Image System (Bio-Rad).
Panel generated from this original image: vinculin of Fig 5b.

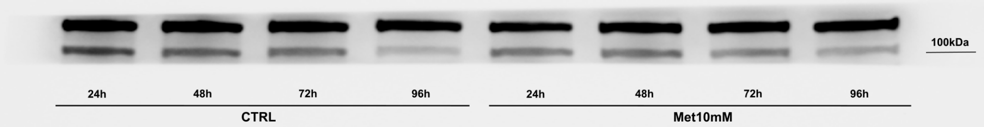

Identity of experimental samples: C2C12 total lysates.
Image captured by using ChemiDoc™ Touch Image System (Bio-Rad).
Panel generated from this original image: ACCß of Fig 5c.

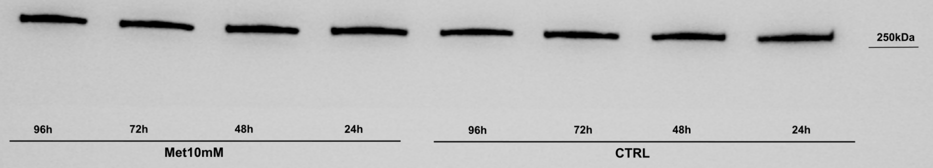

Identity of experimental samples: C2C12 total lysates.
Image captured by using ChemiDoc™ Touch Image System (Bio-Rad).
Panel generated from this original image: p-ACC of Fig 5c.

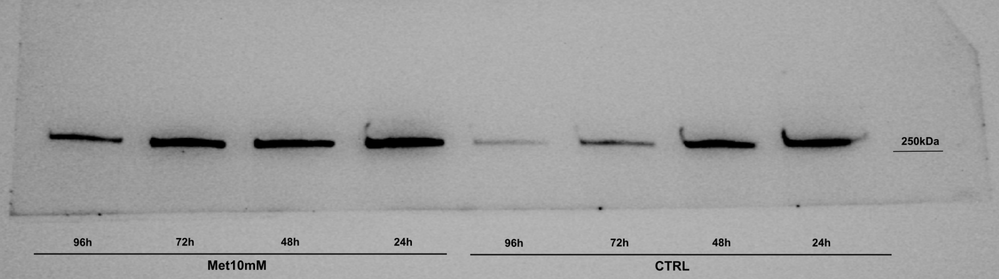

Identity of experimental samples: C2C12 total lysates.
Image captured by using ChemiDoc™ Touch Image System (Bio-Rad).
Panel generated from this original image: GSK3ß of Fig 5d.

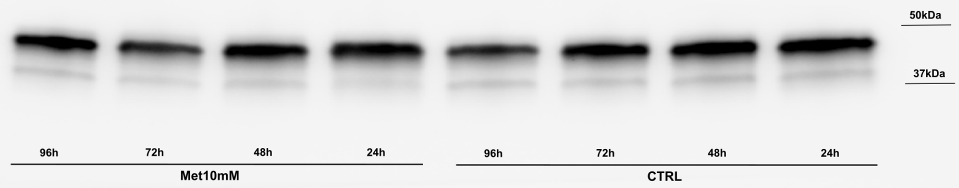

Identity of experimental samples: C2C12 total lysates.
Image captured by using ChemiDoc™ Touch Image System (Bio-Rad).
Panel generated from this original image: p-GSK3ß of Fig 5d.

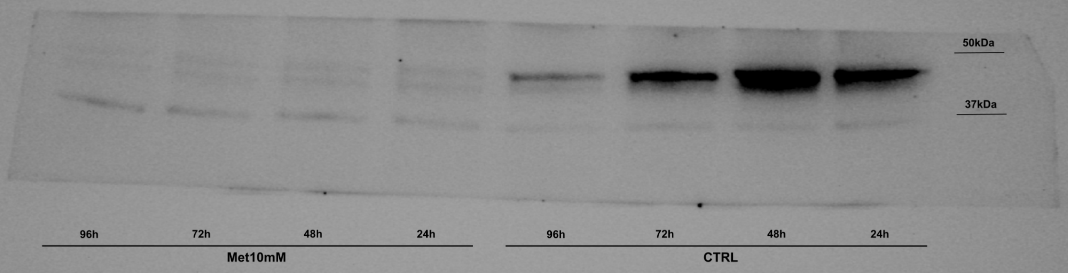

Identity of experimental samples: C2C12 total lysates.
Image captured by using ChemiDoc™ Touch Image System (Bio-Rad).
Panel generated from this original image: MYH1/2 of Fig 6a.

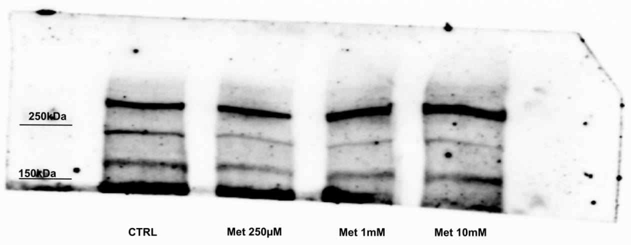

Identity of experimental samples: C2C12 total lysates.
Image captured by using ChemiDoc™ Touch Image System (Bio-Rad).
Panel generated from this original image: GAPDH of Fig 6a.

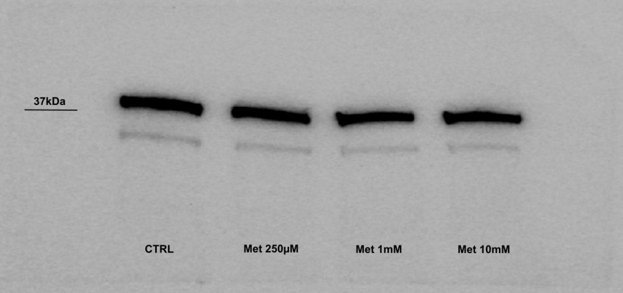

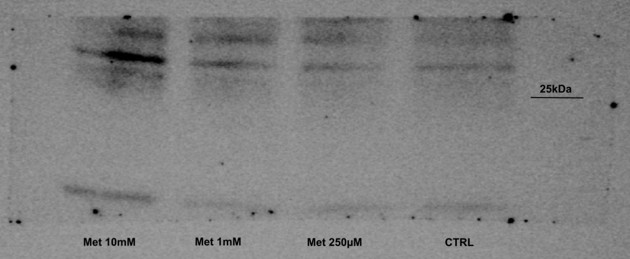

Identity of experimental samples: C2C12 total lysates.
Image captured by using ChemiDoc™ Touch Image System (Bio-Rad).
Panel generated from this original image: Myf5 of Fig 6a.

Identity of experimental samples: C2C12 total lysates.
Image captured by using ChemiDoc™ Touch Image System (Bio-Rad).
Panel generated from this original image: MyoD of Fig 6a.

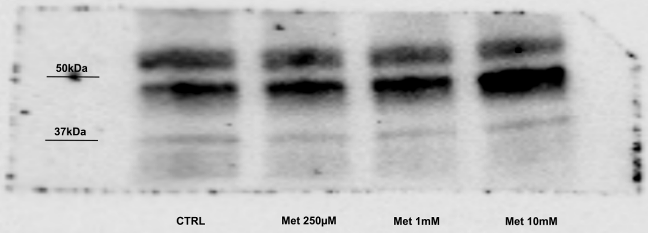

Identity of experimental samples: C2C12 total lysates.
Image captured by using ChemiDoc™ Touch Image System (Bio-Rad).
Panel generated from this original image: GAPDH of Fig 6a.

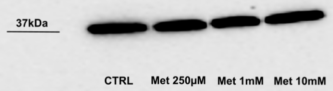

Identity of experimental samples: C2C12 total lysates.
Image captured by using ChemiDoc™ Touch Image System (Bio-Rad).
Panel generated from this original image: p21 of Fig 6a.

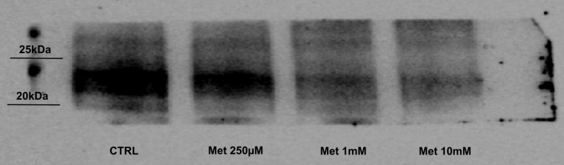

Identity of experimental samples: C2C12 total lysates.
Image captured by using ChemiDoc™ Touch Image System (Bio-Rad).
Panel generated from this original image: GAPDH of Fig 6a.

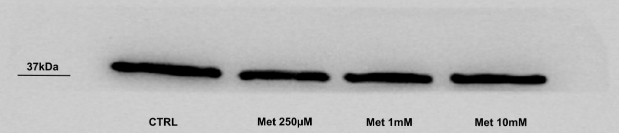

Identity of experimental samples: C2C12 total lysates.
Image captured by using ChemiDoc™ Touch Image System (Bio-Rad).
Panel generated from this original image: AMPK of Fig 7a.

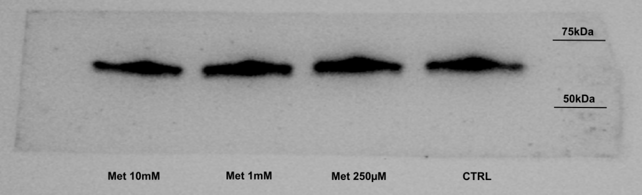

Identity of experimental samples: C2C12 total lysates.
Image captured by using ChemiDoc™ Touch Image System (Bio-Rad).
Panel generated from this original image: p-AMPK of Fig 7a.

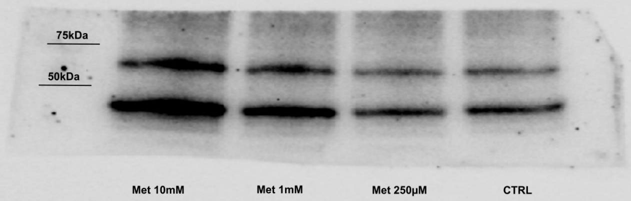

Identity of experimental samples: C2C12 total lysates.
Image captured by using ChemiDoc™ Touch Image System (Bio-Rad).
Panel generated from this original image: PGC1α of Fig 7b.

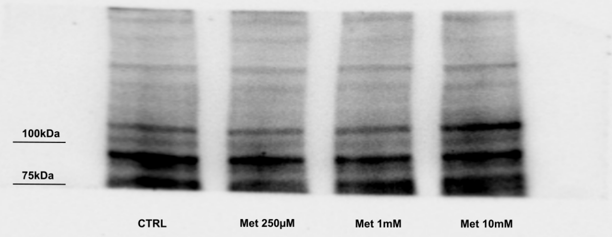

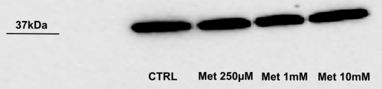

Identity of experimental samples: C2C12 total lysates.
Image captured by using ChemiDoc™ Touch Image System (Bio-Rad).
Panel generated from this original image: GAPDH of Fig 7b.

Identity of experimental samples: C2C12 total lysates.
Image captured by using ChemiDoc™ Touch Image System (Bio-Rad).
Panel generated from this original image: ACCß of Fig 7c.

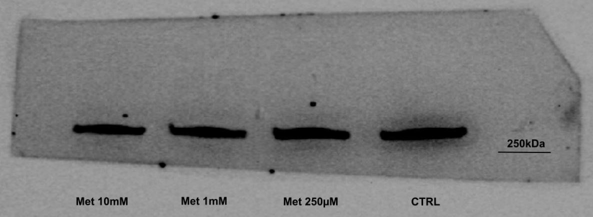

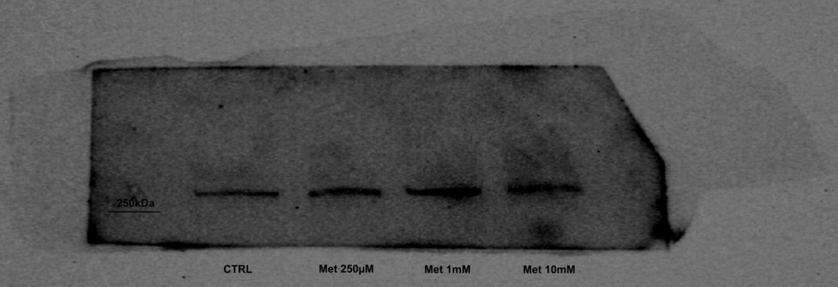

Identity of experimental samples: C2C12 total lysates.
Image captured by using ChemiDoc™ Touch Image System (Bio-Rad).
Panel generated from this original image: p-ACC of Fig 7c.

Identity of experimental samples: C2C12 total lysates.
Image captured by using ChemiDoc™ Touch Image System (Bio-Rad).
Panel generated from this original image: GSK3ß of Fig 7d.

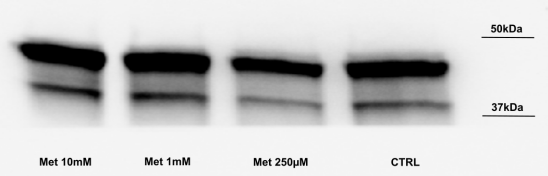

Identity of experimental samples: C2C12 total lysates.
Image captured by using ChemiDoc™ Touch Image System (Bio-Rad).
Panel generated from this original image: p-GSK3ß of Fig 7d.

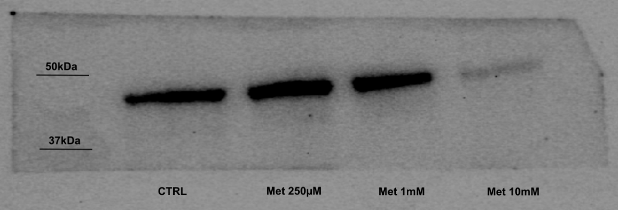

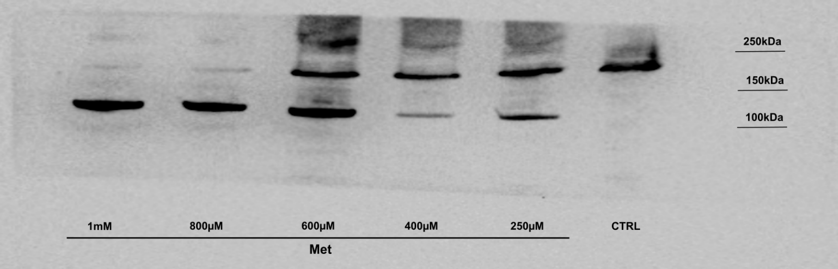

Identity of experimental samples: C2C12 total lysates.
Image captured by using ChemiDoc™ Touch Image System (Bio-Rad).
Panel generated from this original image: MYH1/2 of Fig 8a.

Identity of experimental samples: C2C12 total lysates.
Image captured by using ChemiDoc™ Touch Image System (Bio-Rad).
Panel generated from this original image: Myf5 of Fig 8a.

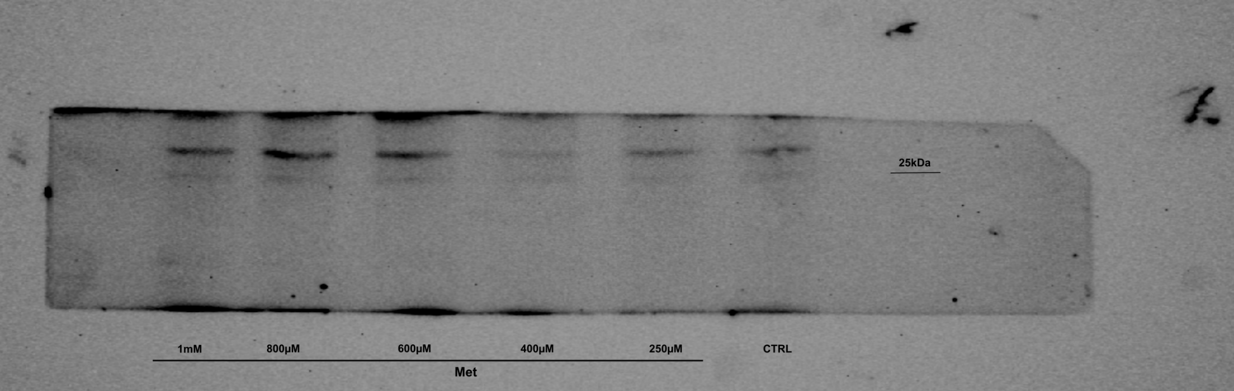

Identity of experimental samples: C2C12 total lysates.
Image captured by using ChemiDoc™ Touch Image System (Bio-Rad).
Panel generated from this original image: p21 of Fig 8a.

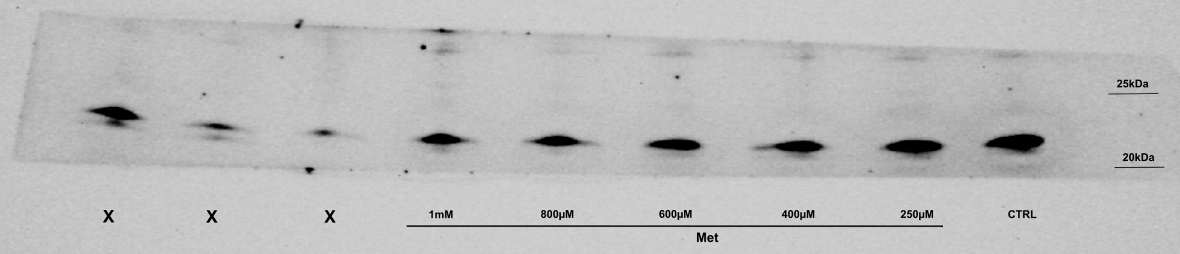

Identity of experimental samples: C2C12 total lysates.
Image captured by using ChemiDoc™ Touch Image System (Bio-Rad).
Panel generated from this original image: PGC1α of Fig 8a.

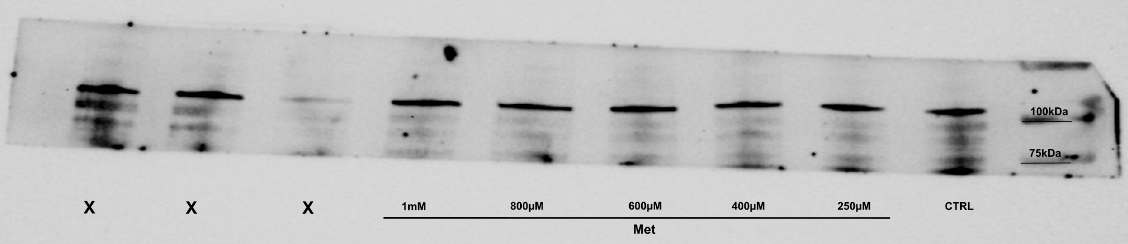

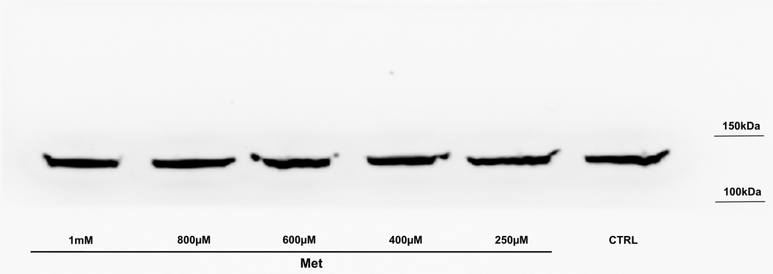

Identity of experimental samples: C2C12 total lysates.
Image captured by using ChemiDoc™ Touch Image System (Bio-Rad).
Panel generated from this original image: vinculin of Fig 8a.

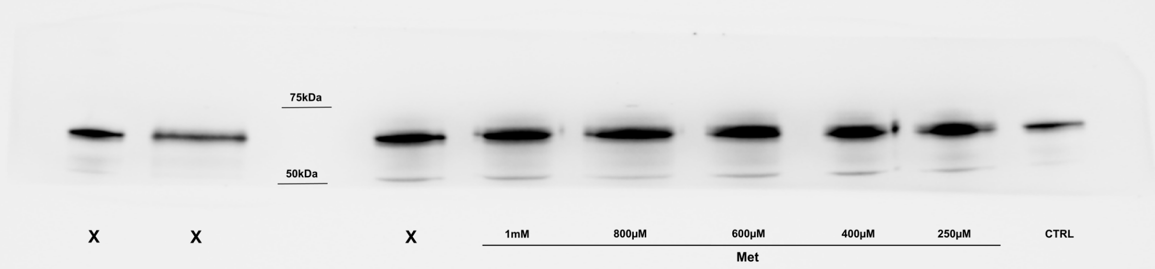

Identity of experimental samples: C2C12 total lysates.
Image captured by using ChemiDoc™ Touch Image System (Bio-Rad).
Panel generated from this original image: AMPK of Fig 9a.

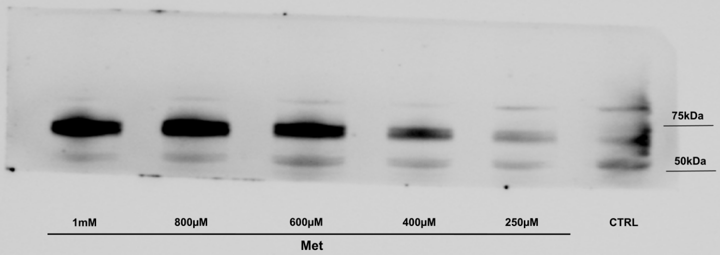

Identity of experimental samples: C2C12 total lysates.
Image captured by using ChemiDoc™ Touch Image System (Bio-Rad).
Panel generated from this original image: p-AMPK of Fig 9a.

Identity of experimental samples: C2C12 total lysates.
Image captured by using ChemiDoc™ Touch Image System (Bio-Rad).
Panel generated from this original image: GSK3ß of Fig 9b.

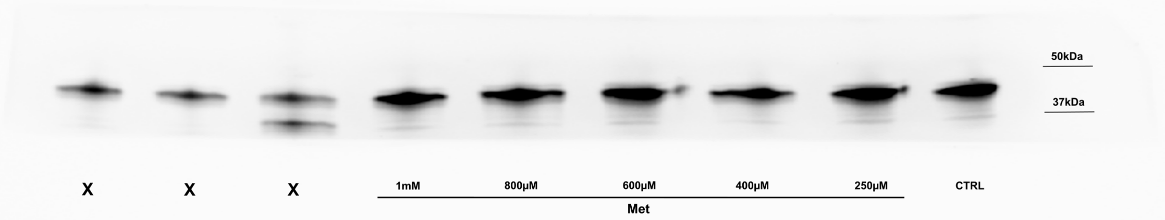

Identity of experimental samples: C2C12 total lysates.
Image captured by using ChemiDoc™ Touch Image System (Bio-Rad).
Panel generated from this original image: p-GSK3ß of Fig 9b.

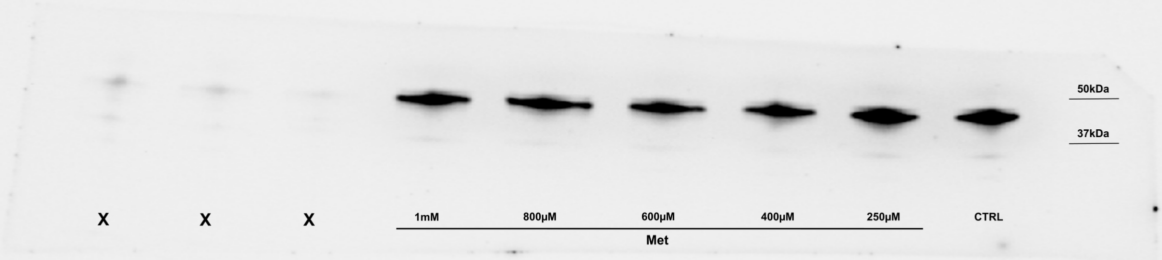

Identity of experimental samples: C2C12 total lysates.
Image captured by using ChemiDoc™ Touch Image System (Bio-Rad).
Panel generated from this original image: MYH1/2 of Fig 11a.

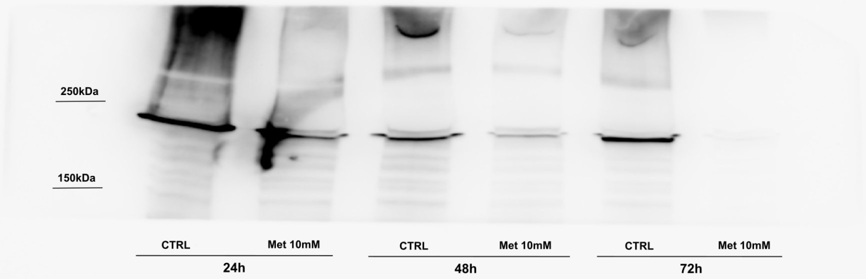

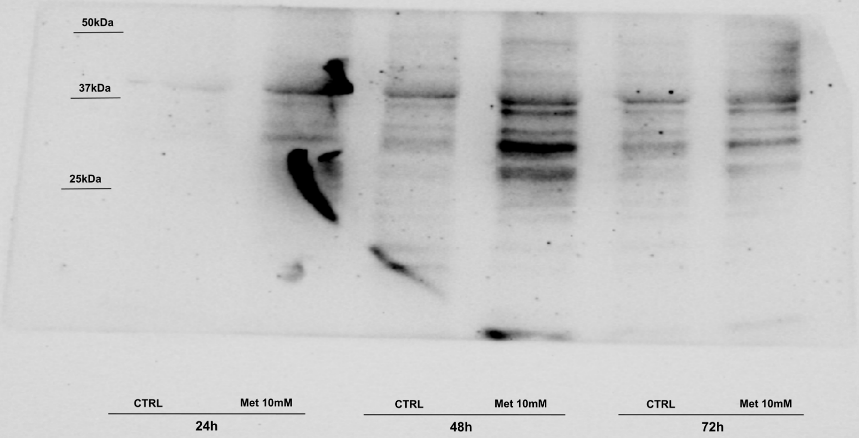

Identity of experimental samples: C2C12 total lysates.
Image captured by using ChemiDoc™ Touch Image System (Bio-Rad).
Panel generated from this original image: Myf5 of Fig 11a.

Identity of experimental samples: C2C12 total lysates.
Image captured by using ChemiDoc™ Touch Image System (Bio-Rad).
Panel generated from this original image: p21 of Fig 11a.

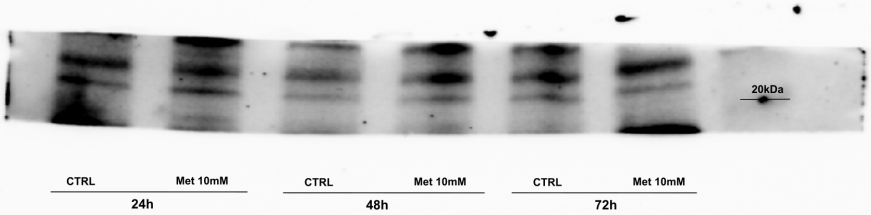

Identity of experimental samples: C2C12 total lysates.
Image captured by using ChemiDoc™ Touch Image System (Bio-Rad).
Panel generated from this original image: PGC1α of Fig 11a.

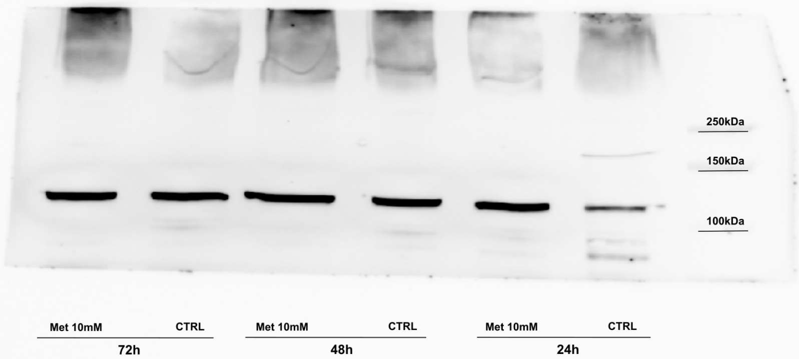

Identity of experimental samples: C2C12 total lysates.
Image captured by using ChemiDoc™ Touch Image System (Bio-Rad).
Panel generated from this original image: vinculin of Fig 11a.

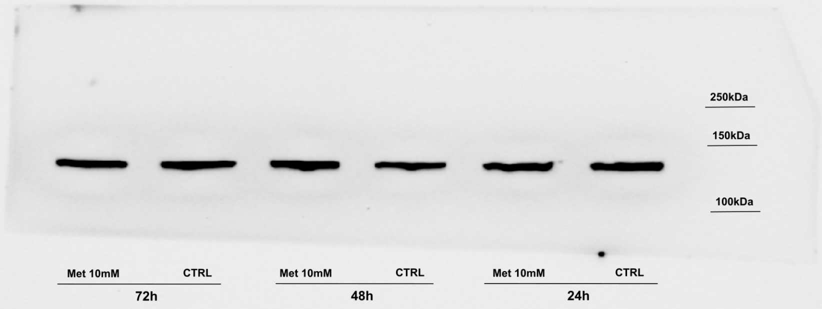

Identity of experimental samples: C2C12 total lysates.
Image captured by using ChemiDoc™ Touch Image System (Bio-Rad).
Panel generated from this original image: AMPK of Fig 12a.

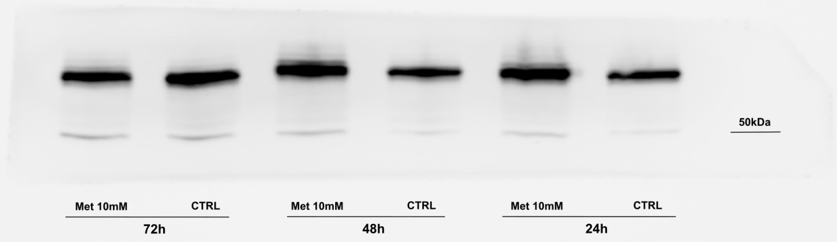

Identity of experimental samples: C2C12 total lysates.
Image captured by using ChemiDoc™ Touch Image System (Bio-Rad).
Panel generated from this original image: p-AMPK of Fig 12a.

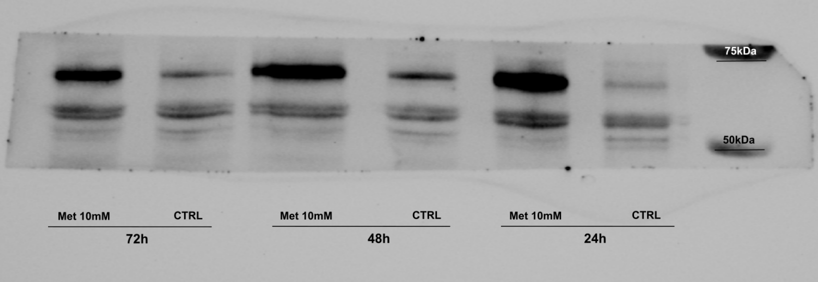

Identity of experimental samples: C2C12 total lysates.
Image captured by using ChemiDoc™ Touch Image System (Bio-Rad).
Panel generated from this original image: GSK3ß of Fig 12b.

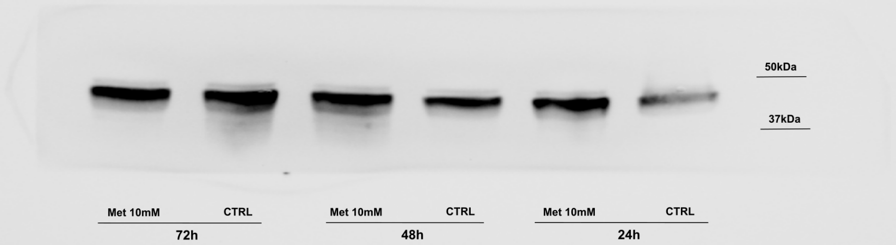

Identity of experimental samples: C2C12 total lysates.
Image captured by using ChemiDoc™ Touch Image System (Bio-Rad).
Panel generated from this original image: p-GSK3ß of Fig 12b.

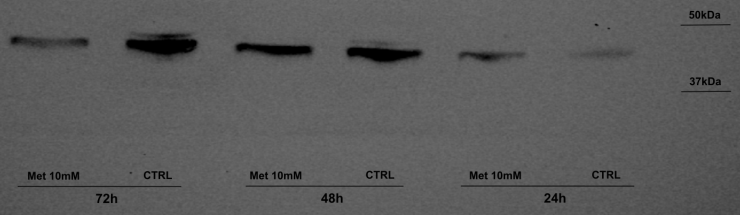

Identity of experimental samples: C2C12 total lysates.
Image captured by using ChemiDoc™ Touch Image System (Bio-Rad).
Panel generated from this original image: MYH1/2 of Fig 13a.

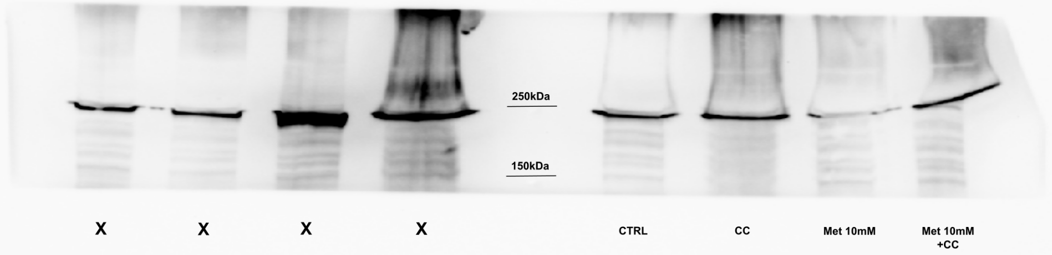

Identity of experimental samples: C2C12 total lysates.
Image captured by using ChemiDoc™ Touch Image System (Bio-Rad).
Panel generated from this original image: Myf5 of Fig 13a.

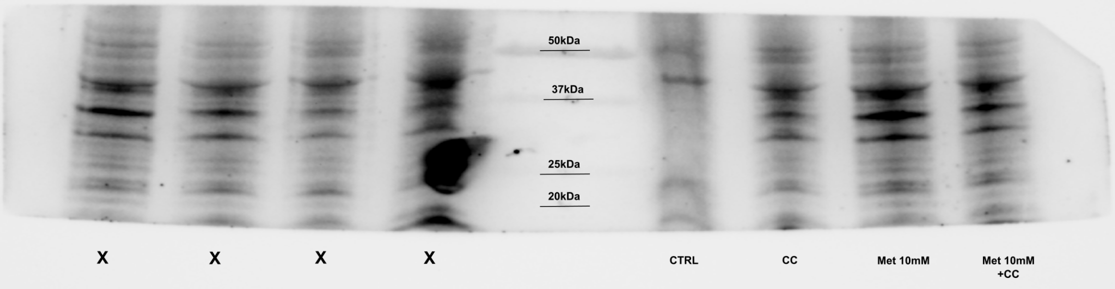

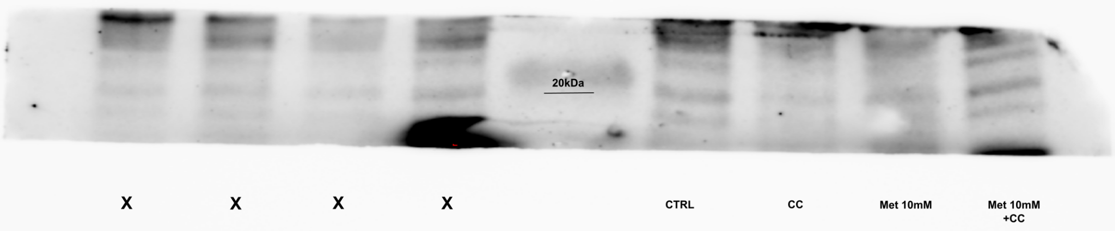

Identity of experimental samples: C2C12 total lysates.
Image captured by using ChemiDoc™ Touch Image System (Bio-Rad).
Panel generated from this original image: p21 of Fig 13a.

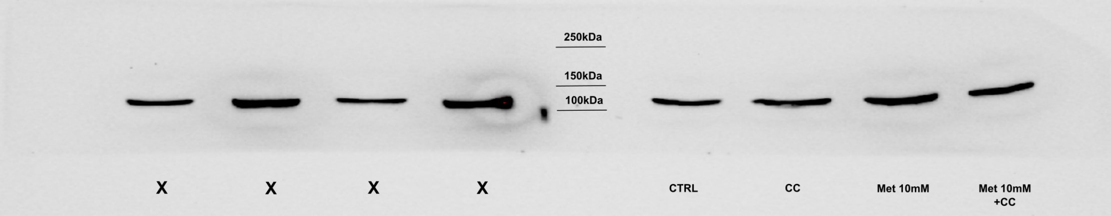

Identity of experimental samples: C2C12 total lysates.
Image captured by using ChemiDoc™ Touch Image System (Bio-Rad).
Panel generated from this original image: vinculin of Fig 13a.

Identity of experimental samples: C2C12 total lysates.
Image captured by using ChemiDoc™ Touch Image System (Bio-Rad).
Panel generated from this original image: PGC1α of Fig 13a.

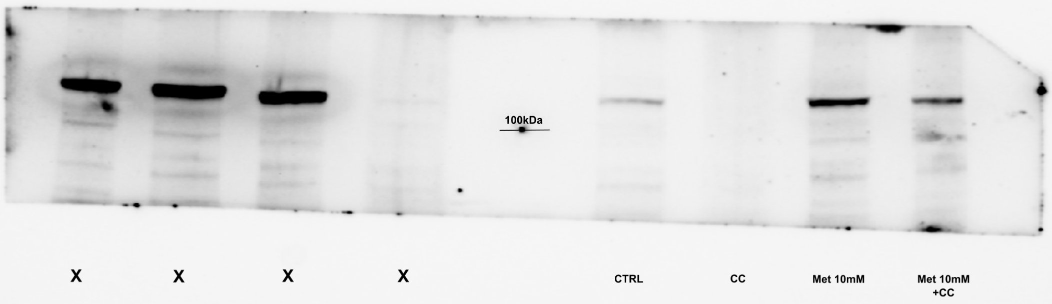

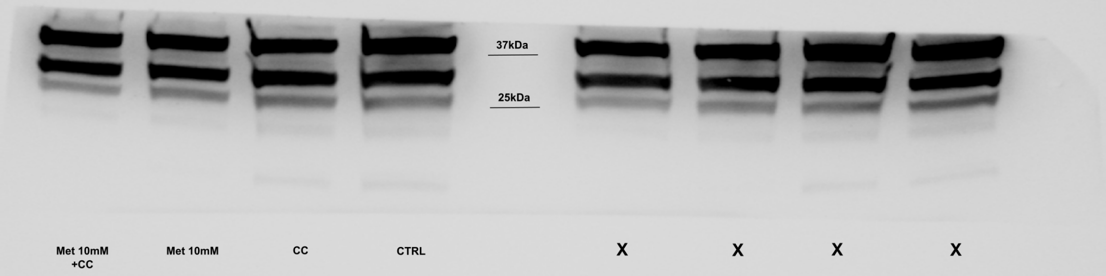

Identity of experimental samples: C2C12 total lysates.
Image captured by using ChemiDoc™ Touch Image System (Bio-Rad).
Panel generated from this original image: GAPDH of Fig 13a.

Identity of experimental samples: C2C12 total lysates.
Image captured by using ChemiDoc™ Touch Image System (Bio-Rad).
Panel generated from this original image: GSk3ß of Fig 13a.

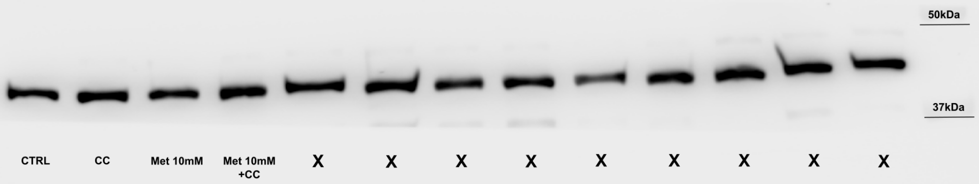

Identity of experimental samples: C2C12 total lysates.
Image captured by using ChemiDoc™ Touch Image System (Bio-Rad).
Panel generated from this original image: p-GSK3ß of Fig 13a.

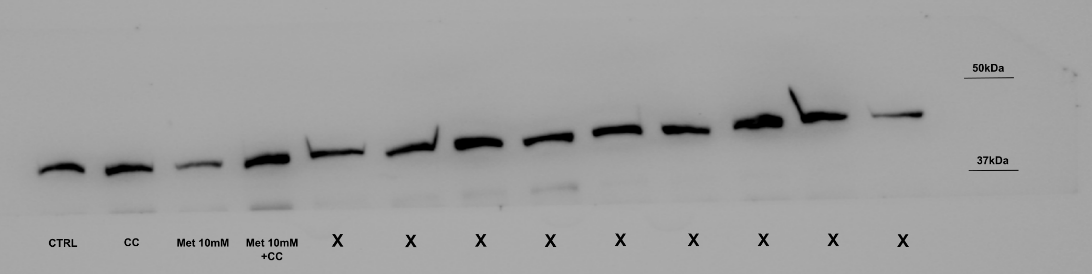

Identity of experimental samples: C2C12 total lysates.
Image captured by using ChemiDoc™ Touch Image System (Bio-Rad).
Panel generated from this original image: MYH1/2 of Fig 14a.

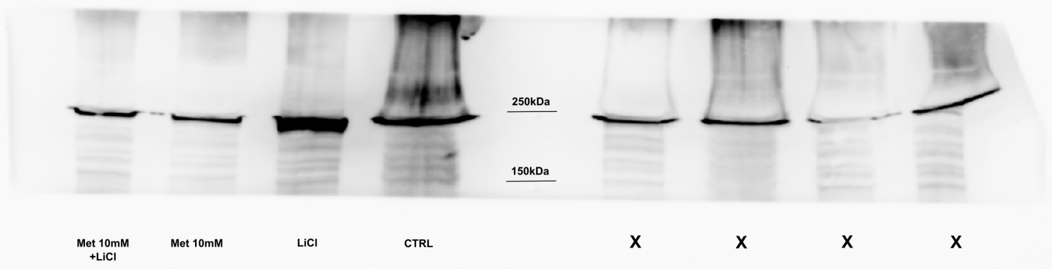

Identity of experimental samples: C2C12 total lysates.
Image captured by using ChemiDoc™ Touch Image System (Bio-Rad).
Panel generated from this original image: Myf5 of Fig 14a.

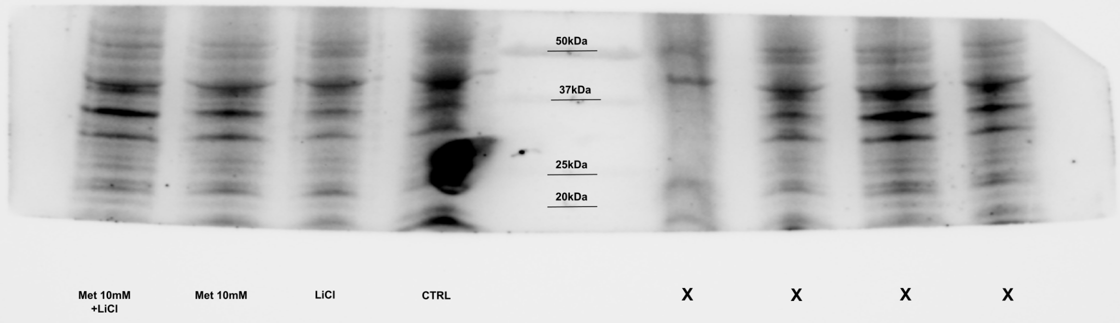

Identity of experimental samples: C2C12 total lysates.
Image captured by using ChemiDoc™ Touch Image System (Bio-Rad).
Panel generated from this original image: PGC1α of Fig 14a.

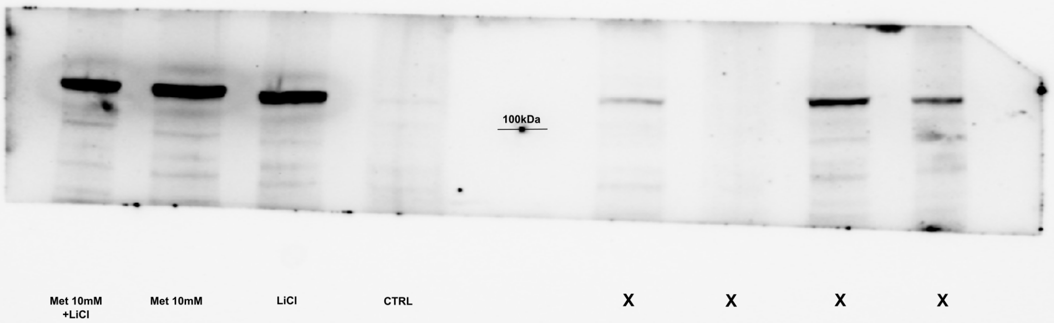

Identity of experimental samples: C2C12 total lysates.
Image captured by using ChemiDoc™ Touch Image System (Bio-Rad).
Panel generated from this original image: vinculin of Fig 14a.

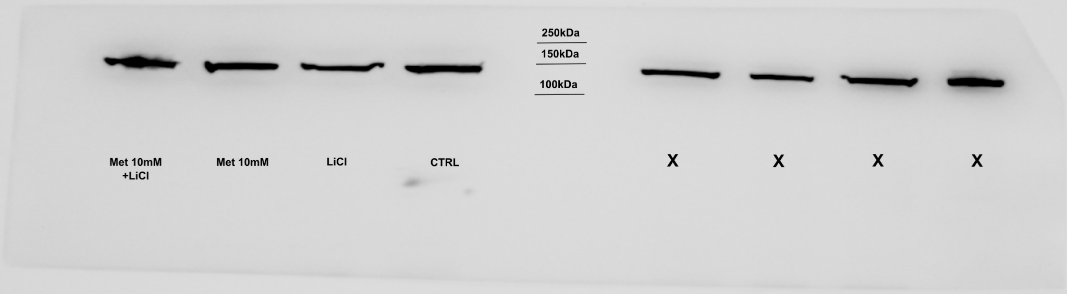

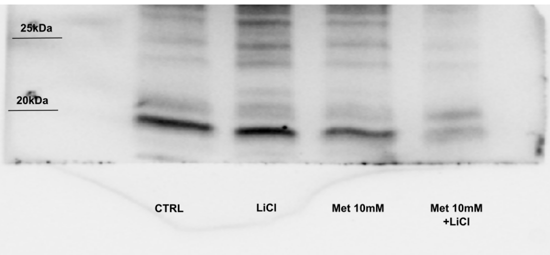

Identity of experimental samples: C2C12 total lysates.
Image captured by using ChemiDoc™ Touch Image System (Bio-Rad).
Panel generated from this original image: p21 of Fig 14a.

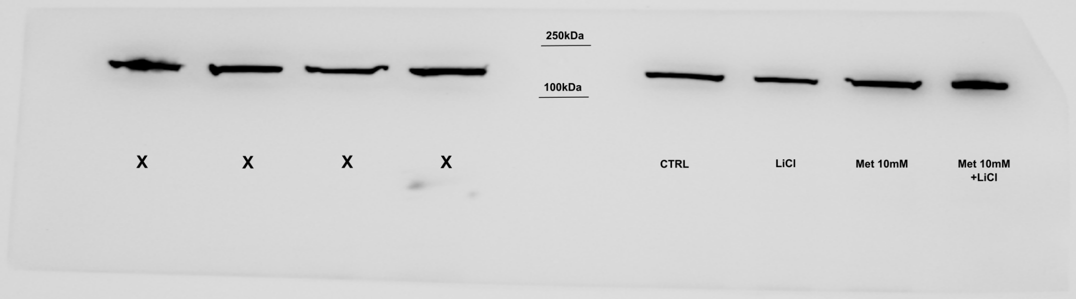

Identity of experimental samples: C2C12 total lysates.
Image captured by using ChemiDoc™ Touch Image System (Bio-Rad).
Panel generated from this original image: vinculin of Fig 14a.
