# Supplementary material for: Metformin regulates myoblast differentiation through an AMPK-dependent mechanism
Source: PLoS One. 2023 Feb 10;18(2):e0281718. doi: 10.1371/journal.pone.0281718 (PMC9916624; doi:10.1371/journal.pone.0281718)
Supplement: Attachment — Submitted filename: Response to Reviewers.docx [file pone.0281718.s005.docx]

Dear Editor,

thank you for the comments regarding our manuscript (PONE-D-17370R1) entitled “Metformin regulates myoblast differentiation through an AMPK-dependent mechanism” by E. Maniscalco et al.

The Reviewer’s remarks spurred us to further improve the overall quality of our research.

Yours faithfully,

Eleonora Maniscalco

Authors’ responses to Reviewer #1:

**Reviewer #1**

*Comment to the Authors:* The manuscript is improved over the previous version. However, the authors need to show whether the molecular effects of Ampk inhibitor treatment are reflected in a recovery of myotubes morphology as well. So the authors need to add the analyses shown in Figure 10 also for myotubes treated with compound C. The housekeeping bands shown in Figure 4b-4c and 4e are the same. The authors should eliminate duplicates and show in one panel the bands revealed on the same filter. The same should be done for the blots in Figure 6b-6c, Figure 6a-6d, Figure 8a-8b-8c-9b, Figure 9a-9c, Figure 11a-11b-11c-12b, Figure 13a-13b-13d, Figure 14a-14b-14d.

**Authors’ response/action:** We agree with the Reviewer’s remarks.

As suggested, we evaluated the effects of the AMPK inhibitor on myotubes at a morphological level. We observed that the molecular effects of compound C are reflected in a recovery of myotubes morphology as well. These analyses have been inserted in the manuscript (Materials and methods, lines 184-186; 190-191; Results, lines 408-409, Figs 10c and 10d; Conclusions, lines 629-630).

As regards the remarks on blot figures, we eliminated the housekeeping duplicates and showed together the bands revealed on the same filter according to the Reviewer’s suggestion (Fig 4, Fig 6, Fig 8, Fig 9, Fig 11, Fig 12, Fig 13, Fig 14 and related captions).
